# HLA class II antigens in Croatian patients with pemphigus vulgaris and their correlation with anti-desmoglein antibodies

**DOI:** 10.3389/fimmu.2023.1200992

**Published:** 2023-07-17

**Authors:** Ines Lakoš Jukić, Mislav Mokos, Branka Marinović

**Affiliations:** ^1^ Department of Dermatovenereology, University Hospital Centre Zagreb, Zagreb, Croatia; ^2^ School of Medicine, University of Zagreb, Zagreb, Croatia

**Keywords:** pemphigus vulgaris, genetics, HLA class II allele, allele frequency, desmoglein

## Abstract

Pemphigus vulgaris (PV) is an acquired autoimmune blistering disease characterized by the production of autoantibodies targeting desmosomal cadherins, primarily desmoglein 1 and desmoglein 3, leading to acantholysis. The etiology of PV is multifactorial, including genetic susceptibility. This retrospective study aimed to evaluate the association of HLA class II alleles and PV and to examine the impact of PV-associated HLA class II alleles on the concentration of anti-desmoglein antibodies. The study group included 30 patients in whom the diagnosis of PV was confirmed by histopathological analysis, immunofluorescence findings, and ELISA testing for detecting antibodies against desmoglein 1 and desmoglein 3. HLA class II alleles were typed by polymerase chain reaction with sequence-specific primers (PCR-SSP). The control group consisted of 190 healthy volunteer blood donors. Data analysis revealed a significantly higher frequency of HLA class II alleles in our population of patients with PV, including HLA-DRB1*04:02, HLA-DRB1*14:54, HLA-DQB1*03:02, HLA-DQB1*05:03, HLA- DQA1*03:01, and HLA-DQA1*01:04, as well as a significantly lower frequency of HLA-DQA1*05:01 compared to the control group. We have also investigated the influence of risk alleles for PV, recognized in almost all study populations, HLA-DRB1*04:02 and HLA-DQB1*05:03, on the concentration of antibodies against desmogleins 1 and 3 in relation to the presence of these alleles. The results showed significantly higher levels of antibodies directed against desmoglein 3 among patients with DRB1*04:02 compared to patients without this allele. No difference was found for anti-desmoglein 1 antibodies. Regarding DQB1*05:03 allele, statistical analysis showed no differences in the concentration of anti-desmoglein antibodies in patients carrying this allele versus those without it.

## 1 Introduction

Pemphigus vulgaris (PV) is a rare disease with an estimated incidence in Croatia of 3.7 new patients per million inhabitants per year (1). Although rare, PV prevalence and incidence differ significantly among different populations, ranging from 0.76 in Finland to 16.1 per million inhabitants in Israel, indicating that genetic factors are involved in the complex pathophysiology of the disease (2, 3). The estimated worldwide incidence is 0.75 to 5 new cases per million annually (4). The highest incidence of 32 new cases was observed in the study of Simon et al. in North America in people of Jewish descent compared to 4.2 new cases in non-Jewish adults (5). PV is more common in the Jewish populations, particularly of Ashkenazi origin, and in the Mediterranean (6, 7).

Hahn-Ristic et al. reported more than an eight-fold higher incidence of PV in foreigners, mainly Turks and Italians, living in Germany than in native Germans (8). The diversity of incidences of PV among different populations and ethnic groups is most likely connected to diverse genetic backgrounds and trigger factors. Population studies have shown a strong association between particular HLA class II alleles and PV. The two most common PV-associated alleles are DQB1*05:03 and DRB1*04:02, as reported in studies from Spain, France, Italy, Slovakia, North America, and Brazil (9–16). According to Kridin et al., most patients with PV express one of these two alleles (17). HLA DRB1*04:02 allele was found in 92% of Ashkenazi Jews patients with PV (18). A statistically significant association was found in the Jewish population with the DQB1*03:02 allele (19). Another DRB1 allele, DRB1*14:01, was positively associated with PV in Japanese, Italian, Pakistani, and Spanish patients (13, 20–22). In white European patients with PV, DRB1*14:54 has been significantly more expressed (16, 23).

The most commonly PV-associated alleles, DQB1*05:03 and DRB1*04:02, are considered to be not only genetic markers for susceptibility but can also influence the severity of the disease. Dhanda et al. have reported that anti-desmoglein 3 autoantibodies levels were significantly higher in patients who carried one or both of these alleles (24).

This study aimed to evaluate the association of HLA class II alleles in our group of patients with PV, compared with healthy subjects, and to determine the influence of PV-associated alleles on the levels of anti-desmoglein antibodies, which correlate with disease severity.

## 2 Materials and methods

### 2.1 Patients and controls

This retrospective study sampled 30 unrelated patients (20 female and 10 male) treated for PV at the Department of Dermatovenereology, University Hospital Centre Zagreb. The mean age of patients was 54 years, with a range of 33-82 years. The diagnosis of PV was based on clinical, histological, and immunofluorescence studies and ELISA testing to detect serum antibodies against Dsg1 and Dsg3 (MESACUP Desmoglein test, Nagano, Japan). Additionally, serum samples were collected for HLA typing, for which all patients signed informed consent. The control group consisted of 190 (41 female and 149 male) healthy volunteer blood donors with a mean age of 40 years and a range of 18-71 years. The study protocol has been approved by the Committee of Ethics of the University Hospital Centre Zagreb and was conducted in accordance with the Declaration of Helsinki.

### 2.2 DNA extraction and HLA typing

Genomic DNA was extracted from peripheral blood (3 ml) using QIAamp DNA Blood mini kit (Qiagen, Hilden, Germany). HLA class II alleles were typed by polymerase chain reaction with sequence-specific primers (PCR-SSP). Detection of products of PCR amplification was performed by electrophoresis on 1.5% agarose gel. Gel images were analyzed with standardized tables or with HELMBERG SCORE software.

### 2.3 Statistical analysis

The frequencies of HLA alleles were directly extracted from the genotyping data. The statistical analysis was performed using the STATISTICA software package version 12 (www.statsoft.com. StatSoft, Inc. Tulsa, OK, USA) and the VassarStats website for Statistical Computation (http://vassarstats.net/odds2x2.html). The categorical (qualitative) variables were presented as frequencies and percentages and compared between groups (PV and control) using Fisher’s exact test. The normality of antibody concentration distribution was assessed using the Kolmogorov-Smirnov test, and antibody concentrations were presented as the median, interquartile range (IQR), and range. The comparison between groups regarding the presence of HLA DRB1*04:02 and/or HLA DQB1*05:03 was performed using the Mann-Whitney U test. The association between HLA alleles and PV was determined using the odds ratio (OR) and 95% confidence intervals (95%CI). The Benjamini-Hochberg method was used to adjust for multiple comparisons. A two-tailed P-value (or adjusted P-value) <0.05 was considered statistically significant.

## 3 Results

The frequencies of HLA-DRB1 allele in pemphigus patients and control group are shown in **Table 1**. The presence of DRB1*04:02 (OR=24.69; 95%CI=9.80-62.25; P=2.55*10^-11^) and DRB1*14:54 (OR=27.07; 95%CI=3.35-218,77; P=0.0011) was significantly higher in group of PV patients compared to controls. There were no significant differences for other alleles in DR region.

**Table 1:** Frequencies of HLA-DRB1 alleles in patients with PV and control group.

	PV		Control						
*DRB1*	N	%	N	%	*P*	*P_adj_*	OR	-95%CI	+95%CI
*0402	**19**	**31,67**	**7**	**1,84**	**7,29x10^-13^**	**2,55x10^-11^**	**24,693**	**9,795**	**62,252**
*1104	6	10,00	29	7,63	0,605496	1	1,345	0,534	3,390
*1601	6	10,00	37	9,74	1	1	1,030	0,415	2,557
*1401	4	6,67	13	3,42	0,268546	0,854465	2,017	0,635	6,403
*1454	**4**	**6,67**	**0**	**0,00**	**6,59E-05**	**0,001153**	**27,071**	**3,350**	**218,773**
*1101	3	5,00	38	10,00	0,245455	0,854465	0,474	0,142	1,586
*1602	3	5,00	6	1,58	0,11095	0,63188	3,281	0,798	13,487
*0101	3	5,00	36	9,47	0,333431	0,972506	0,503	0,150	1,688
*0701	3	5,00	24	6,32	0,784735	1	0,781	0,228	2,677
*0401	2	3,33	13	3,42	1	1	0,991	0,218	4,505
*1501	1	1,67	34	8,95	0,068093	0,595815	0,173	0,023	1,284
*1301	1	1,67	24	6,32	0,228298	0,854465	0,251	0,033	1,894
*0801	1	1,67	13	3,42	0,703748	1	0,479	0,061	3,726
*1404	1	1,67	0	0,00	0,136364	0,63188	Inf	NA	Inf
*0804	1	1,67	6	1,58	1	1	1,057	0,125	8,933
*0302	1	1,67	0	0,00	0,136364	0,63188	Inf	NA	Inf
*1201	1	1,67	8	2,11	1	1	0,788	0,097	6,417
*0102	0	0,00	5	1,32	0,61804	1	0	0	NA
*0103	0	0,00	3	0,79	1	1	0	0	NA
*0301	0	0,00	37	9,74	0,009396	0,109621	0	0	NA
*0403	0	0,00	2	0,53	1	1	0	0	NA
*0404	0	0,00	7	1,84	0,60062	1	0	0	NA
*0405	0	0,00	1	0,26	1	1	0	0	NA
*0407	0	0,00	1	0,26	1	1	0	0	NA
*0812	0	0,00	1	0,26	1	1	0	0	NA
*1001	0	0,00	6	1,58	0,603815	1	0	0	NA
*1103	0	0,00	3	0,79	1	1	0	0	NA
*1111	0	0,00	1	0,26	1	1	0	0	NA
*1302	0	0,00	16	4,21	0,14443	0,63188	0	0	NA
*1303	0	0,00	2	0,53	1	1	0	0	NA
*1305	0	0,00	1	0,26	1	1	0	0	NA
*1405	0	0,00	1	0,26	1	1	0	0	NA
*1408	0	0,00	1	0,26	1	1	0	0	NA
*1502	0	0,00	3	0,79	1	1	0	0	NA
*0901	0	0,00	1	0,26	1	1	0	0	NA
**Total**	**60**	**100,00**	**380**	**100,00**					

N, frequency of allele; %, percentage; P, P value; P_adj_, adjusted P value; OR, odds ratio; Cl, confidence interval; NA, not available; inf, infinite. Alleles with significant frequency differences between the examined groups have been highlighted in bold.

HLA-DQB1 typing results of PV patients and the control group are presented in **Table 2**. Frequencies of DQB1*03:02 (OR=8.81; 95%CI=4.46-17.42; Padj=3.76x10^-8^) and DQB1*05:03 (OR=5.19; 95%CI=2.11-12.76; Padj=0.0068) were significantly increased in PV patients versus controls.

**Table 2:** Frequencies of HLA-DQB1 alleles in patients with PV and control group.

	PV		Control						
*DQB1*	N	%	N	%	*P*	*P_adj_*	OR	-95%CI	+95%CI
*0302	**21**	**36,21**	**23**	**6,05**	**2,35x10^-9^**	**3,76x10^-8^**	**8,809**	**4,456**	**17,417**
*0503	**9**	**15,52**	**13**	**3,42**	**0,000853**	**0,006821**	**5,185**	**2,107**	**12,762**
*0502	9	15,52	42	11,05	0,376976	0,502635	1,478	0,678	3,224
*0301	8	13,79	93	24,47	0,093052	0,248139	0,494	0,226	1,079
*0501	3	5,17	51	13,42	0,086506	0,248139	0,352	0,106	1,17
*0402	2	3,45	12	3,16	1	1	1,095	0,239	5,024
*0303	2	3,45	5	1,32	0,234091	0,374546	2,679	0,507	14,140
*0602	1	1,72	33	8,68	0,067471	0,248139	0,185	0,025	1,376
*0603	1	1,72	26	6,84	0,154532	0,309065	0,239	0,032	1,795
*0321	1	1,72	0	0,00	0,13242	0,302675	Inf	NA	Inf
*0202	1	1,72	20	5,26	0,335955	0,488662	0,316	0,042	2,399
*0201	0	0,00	39	10,26	0,010321	0,055045	0	0	NA
*0304	0	0,00	4	1,05	1	1	0	0	NA
*0307	0	0,00	2	0,53	1	1	0	0	NA
*0601	0	0,00	3	0,79	1	1	0	0	NA
*0604	0	0,00	14	3,68	0,232458	0,374546	0	0	NA
**Total**	**58**	**100,00**	**380**	**100,00**					

N, frequency of allele; %, percentage; P, P value; P_adj_, adjusted P value; OR, odds ratio; Cl, confidence interval; NA, not available; inf, infinite. Alleles with significant frequency differences between the examined groups have been highlighted in bold.

As shown in **Table 3**, within HLA-DQA1* locus, a significantly higher frequency of the HLA-DQA1*03:01 allele (OR=7.76; 95%CI=3.88-15.53; Padj=6.66x10^-7^) and HLA-DQA1*01:04 allele (OR=4.27; 95%CI=1.79-10.19; Padj=0.01198) were found among PV patients, compared to the control group. The frequency of DQA1*05:01 allele was significantly decreased in PV group versus the control (OR=<0.01; 95%CI=<0.001-<0.05; Padj=0,00226).

**Table 3:** Frequencies of HLA-DQA1 alleles in patients with PV and control group.

	PV		Control						
*DQA1*	N	%	N	%	*P*	*P_adj_*	OR	-95%CI	+95%CI
*0301	**19**	**33,33**	**23**	**6,05**	**4,16x10^-8^**	**6,66x10^-7^**	**7,761**	**3,879**	**15,529**
*0102	9	15,79	95	25,00	0,137081	0,438659	0,563	0,266	1,189
*0104	**9**	**15,79**	**16**	**4,21**	**0,002247**	**0,011981**	**4,266**	**1,786**	**10,185**
*0505	8	14,04	40	10,53	0,493584	0,987167	1,388	0,614	3,139
*0101	3	5,26	42	11,05	0,24322	0,648587	0,447	0,134	1,493
*0201	3	5,26	24	6,32	1	1	0,824	0,240	2,830
*0103	2	3,51	26	6,84	0,406944	0,930158	0,495	0,114	2,145
*0401	2	3,51	14	3,68	1	1	0,951	0,210	4,297
*0402	1	1,75	0	0,00	0,130435	0,438659	Inf	NA	Inf
*0303	1	1,75	10	2,63	1	1	0,661	0,083	5,261
*0105	0	0,00	6	1,58	0,60776	1	0	0	NA
*0302	0	0,00	1	0,26	1	1	0	0	NA
*0501	**0**	**0,00**	**80**	**21,05**	**0,000283**	**0,00226**	**<0.01**	**<0.001**	**<0.05**
*0502	0	0,00	1	0,26	1	1	0	0	NA
*0503	0	0,00	1	0,26	1	1	0	0	NA
*0601	0	0,00	1	0,26	1	1	0	0	NA
**Total**	**57**	**100,00**	**380**	**100,00**					

N, frequency of allele; %, percentage; P, P value; P_adj_, adjusted P value; OR, odds ratio; Cl, confidence interval; NA, not available; inf, infinite. Alleles with significant frequency differences between the examined groups have been highlighted in bold.

The association between concentration of anti-desmoglein 3 (anti-Dsg3) and anti-desmoglein 1 (anti-Dsg1) antibodies in relation to the presence or absence of DRB1*04:02 allele is presented in **Table 4**. There was a significantly increased concentration of anti-Dsg3 antibodies in patients who had DRB1*04:02 compared to patients without it (Mann-Whitney test; z=-3.701; p=0.00). No difference was observed for anti-Dsg1 antibodies. **Table 5** shows the association of DQB1*05:03 allele and anti-Dsg3 and anti-Dsg1 antibodies. Statistical analysis showed no difference in the concentration of anti-Dsg1 and anti-Dsg3 antibodies in patients who carry this allele versus those without it.

**Table 4:** The concentration of anti-Dsg1 and anti-Dsg3 antibodies in correlation with the presence or absence of DRB1*04:02 allele.

antibody	DRB1*04:02	N	Median	Minimum	Maximum	IQR
anti-Dsg1	NEG	11	36,52	1,27	168,83	12,05-79.90
anti-Dsg1	POS	19	29,92	0,42	156,71	11,42-92,30
anti-Dsg3	NEG	11	76,62	0,92	156,71	2,49-25,76
anti-Dsg3	POS	19	180,68	38,42	298,92	148,93-201,22

N, number of patients with DRB1*04:02 allele; IQR, interquartile range.

**Table 5:** The concentration of anti-Dsg1 and anti-Dsg3 antibodies in correlation with the presence or absence of DQB1*05:03 allele.

antibody	DQB1*05:03	N	Median	Minimum	Maximum	IQR
anti-Dsg1	NEG	21	35,45	1,38	168,83	17,95-92,30
anti-Dsg1	POS	9	22,55	0,42	150,23	1,27.56,62
anti-Dsg3	NEG	21	156,71	0,92	298,92	91,28-196,83
anti-Dsg3	POS	9	131,85	34,66	185,98	60,50-153,63

N, number of patients with DQB1*05:03 allele; IQR, interquartile range.

## 4 Discussion

Pemphigus vulgaris is a chronic autoimmune bullous disease clinically characterized by erosions on the skin and mucous membranes and immunologically by autoantibodies targeting transmembrane components of desmosome, mainly desmogleins 1 and 3. Even though PV is a rare disease, its pathogenesis is constantly in the focus of research in dermatology, both due to its severe clinical course and significant impact on quality of life.

Different data on the prevalence of PV in relation to gender are reported in the literature. Polman et al. reported an equal representation of genders in PV patients, which is inconsistent with the fact that autoimmune diseases are more common among women (25, 26). Even more, the male preponderance has been reported in two reports from Kuwait and Saudi Arabia (27, 28). In this study, the sample included 20 women and 10 men, corresponding to 2:1 ratio, what is consistent with the recent report regarding the epidemiology of PV, which shows a female predominance with the female-to-male ratio ranging between 1 and 2, with 5 in the study of Simon et al. (5, 17). It is also expected, given the autoimmune nature of PV.

PV is a multifactorial disease resulting from the interaction of genetic and environmental factors (29). Although the predisposition to PV is genetically determined, the way of inheritance is still not clear (19). There are reports about the familial aggregation of the disease, but they are only sporadic, indicating PV is not inherited by a Mendelian pattern (29–32). Still, the presence of anti-Dsg3 antibodies in healthy first-degree relatives of PV patients points to dominant inheritance (29, 33, 34).

The association between PV occurrence and specific HLA antigens has been observed for almost sixty years, with the first report of Krain et al. (35). The role of the HLA system in the pathogenesis of PV corresponds with its direct involvement in immune reactions.

When comparing the frequency of HLA class II alleles in different populations, in groups of PV patients to healthy individuals, a significantly higher frequency of multiple alleles was observed among PV patients. In a meta-analysis of 18 studies published about the association of PV and DRB1* locus, DRB1*04, DRB1*08, and DRB1*14 were significantly overexpressed (36). From all of these, the importance of DRB1*04:02 is by far the greatest. Namely, a significantly higher frequency of this allele has been demonstrated among PV patients compared to healthy individuals in almost all populations (9). The data from our study is in accordance with the aforementioned; a significantly higher frequency of the DRB1*04:02 allele (OR=24.69) was observed in the group of patients with PV compared to the control group. The frequency of this allele within a locus was 31.67% in the patient group, while the same allele had a frequency of 1.84% among healthy individuals. Since there were no patients homozygous for this allele in our study, the frequency of DRB1*04:02 per patient was 63.3%. This result is similar to the recently published report of 293 patients with PV, of which 60% carried DRB1*04:02 allele (37). We have also observed a significantly increased frequency of DRB1*14:54 allele in the PV group (OR=27.071). The association between this allele and PV has been reported in white European individuals in the United Kingdom as well as in Slovakia (16, 23). The association of DRB1*14:54 allele with PV in other populations is probably underreported because previously PV-associated allele DRB1*14:01 may actually represent DRB1*14:54 (10, 12, 20, 38). These alleles differ only in exon 3, at position 112, due to amino replacement (23). In Brazilian patients with PV, there is an increased frequency of the HLA-DRB1*08:04 allele compared to the control group. Since this allele was associated in haplotypes with alleles that do not carry a risk for PV, it was concluded HLA-DRB1*08:04 allele is associated with PV (39). Increased frequency of this allele has also been reported in African American patients with PV (37).

Within the HLA-DQB1* gene, the association of HLA-DQB1*05:03 and DQB1*03:02 alleles with PV has been confirmed by a meta-analysis of 18 population studies (40). However, due to strong linkage disequilibrium between DR and DQ loci, such as the association of DRB1*14:01 and DQB1*05:03 or DRB1*04 and DRB1*03, the question arises as to which of these alleles represents a risk for PV. In their study, Lee et al. found that haplotypes DRB1*04:02-DQB1*03:02 and DRB1*04:02 without DQB1*03:02 are more frequently expressed in patients with PV (14). After separating patients carrying the HLA-DRB1*04:02 allele, they did not find a significant difference in the frequency of the DQB1*03:02 allele. A similar finding was obtained in the study by Saenz-Cantele et al. among PV patients in Venezuela (41). Lee et al. also observed that HLA-DRB1*14:01 alone does not carry a risk for PV but is often found together with HLA-DQB1*05:03 due to the imbalance of association (14). Nowadays, HLA-DRB1*04:02 and HLA-DQB1*05:03 are considered to be the key alleles directly involved in the immunopathogenesis of PV (17, 36, 40). Veldman et al. reported that only antigen-presenting cells expressing HLA-DRB1*04:02 and HLA-DQ1*05:03 could present desmoglein 3 to autoreactive CD4+ T lymphocytes (42). Among our participants, we found a significantly higher frequency of the HLA-DQB1*03:02 allele (OR=8.81) and HLA-DQB1*05:03 allele (OR=5.19) in patients with PV compared to the control group. The higher frequency of DQB1*03:02 can be explained as a result of linkage disequilibrium with DRB1*04:02. HLA-DQB1*05:03 allele was seen in 9 of our PV patients with a frequency of 30% per patient. The same result has been reported in the study of Baker at al. (37). In that study this allele was predominant in the South Asian population with PV; 82% of them carried DQB1*05:03. No significant difference in distribution was found for other alleles of the HLA-DQB1* locus.

In our study, a significantly higher frequency of the HLA-DQA1*03:01 allele (OR=7.76) and HLA-DQA1*01:04 allele (OR=4.27) within the HLA-DQA1* locus was found among PV patients, compared to the control group. This result aligns with similar studies conducted in Iran and Brazil (39, 43). Given that loci DRB1* and DQB1* are in the focus of research due to their role in the pathogenesis of PV, there are scarce literary reports on the association of DQA1* locus genes and PV. Our study demonstrated a significantly lower frequency of the HLA-DQA1*05:01 allele (OR=<0.01) among PV patients compared to the control group. This result suggests a possible protective role of this allele in terms of PV occurrence. A negative association of this allele with psoriasis was also described (44).

The clinical presentation of PV is diverse, depending on the affection of mucous membranes and skin, the course of the disease, the number of lesions, and the effectiveness of treatment. The course of the disease cannot be predicted. In the study by Dhandha et al., patients with mucous membrane lesions and those with both mucous membrane and skin lesions had significantly higher levels of antibodies directed against Dsg3 than patients with only skin changes. Further research reported that the levels of these antibodies were significantly higher in patients who had either the HLA-DRB1*04:02 or HLA-DQB1*05:03 allele (24). The report of Svecova et al. documented a correlation between HLA-DRB1*04:02 and severe mucocutaneous PV (45). HLA-DQB1*03:02 was associated with a more severe form of PV only among female patients. The role of HLA-DRB1*04:02 or HLA-DQB1*05:03 allele in pathophysiology of PV has been additionally highlighted in studies focused on Dsg3 specific CD4 T lymphocyte response, restricted by these two alleles (46–50). Eming et al. demonstrated in an experiment with an HLA- DRB1*04:02 transgenic mouse model that this antigen is involved in activating specific auto-aggressive CD4+ T lymphocytes (50). These lymphocytes have a crucial role in the induction and maintenance of B lymphocytes or plasma cells, producing antibodies directed against Dsg3. Such a T lymphocyte response in patients with PV has been demonstrated in several studies (42, 51, 52). Based on the above, PV can be considered a T-cell, more precisely Th2, mediated autoimmune disease, which certainly opens new treatment possibilities (50).

In our study, we have demonstrated significantly higher levels of antibodies directed against desmoglein 3 in patients who have the HLA- DRB1*04:02 allele compared to those who do not have this allele. There was no difference in the concentration of anti-Dsg1 antibodies. Regarding DQB1*05:03 allele, statistical analysis showed no differences in the concentration of anti-desmoglein antibodies in patients carrying this allele versus those without it. Baker et al. reported higher levels of anti-Dsg1 antibodies in patients carrying DQB1*05:03 allele compared to DRB1*04:02 and DRB1*08:04 positive patients (37). Our result may be the consequence of the small sample size since DQB1*05:03 was expressed in only 9 patients. Given the literature reports of a correlation between antibody concentration and clinical presentation, and the fact that desmoglein 3 is the main autoantigen in PV, it is reasonable to conclude that HLA- DRB1*04:02 may impact the disease severity in our study population (53–55). Unfortunately, due to our study’s retrospective nature, we could not perform further analysis of disease severity grading (ABSIS - Autoimmune Bullous Skin Disorder Intensity Score) in our patients. Lastly, 4 of our 30 patients did not express DRB1*04:02 or DQB1*05:03. Since these alleles have a crucial role in initiating an autoimmune response against desmogleins, further studies will be needed to elucidate the role of other, non-desmoglein autoantibodies in pathophysiology of PV.

## 5 Conclusion

Pemphigus vulgaris is probably a polygenic disease whose phenotype results from complex interactions between more than one gene and environmental factors. It is reasonable to assume that HLA class II genes, especially DRB1*04:2 and DQB1*05:03 are just one piece of the complex puzzle of PV pathophysiology. To the best of our knowledge, this is the first study investigating the association of specific HLA class II alleles with the occurrence of PV in the Republic of Croatia. Through data analysis, we found a significantly higher frequency of HLA class II alleles in our population of patients with PV, including HLA- DRB1*04:02, HLA-DRB1*14:54, HLA-DQB1*03:02, HLA-DQB1*05:03, HLA- DQA1*03:01, and HLA-DQA1*01:04, as well as a significantly lower frequency of HLA-DQA1*05:01 compared to the control group. The significance of this “negatively” associated allele with PV occurrence is unknown. Regarding the fact that pemphigus vulgaris-relevant HLA alleles are restricted to the DRB and DQB loci and strong linkage disequilibrium of DRB1*04:02 with DQB1*03:02 and of DQB1*05:03 with DRB1*14:54, we can conclude that DRB1*04:02 and, to a lesser extent, DQB1*05:03 are risk alleles for PV in Croatia. We have also demonstrated significantly higher levels of antibodies directed against desmoglein 3 in patients with the HLA- DRB1*04:02 compared to those who do not have this allele. Our findings support the previously reported hypothesis that HLA-DRB1*04:02 allele is not only genetic marker for development of PV, but can also influence activity and severity of the disease.

## Data availability statement

The data analyzed in this study is subject to the following licenses/restrictions: They are archived in the hospital’s patient databases. Requests to access these datasets should be directed to branka.marinovic@kbc-zagreb.hr.

## Ethics statement

The studies involving human participants were reviewed and approved by Committee of Ethics of the University Hospital Centre Zagreb. The patients/participants provided their written informed consent to participate in this study.

## Author contributions

ILJ, MM, and BM contributed actively to the preparation of the manuscript. Conceptualization: ILJ; Writing - original draft preparation: ILJ, MM, and BM; Writing - review and editing: ILJ, MM, and BM; Tables: ILJ; Collecting sources of information: ILJ and BM; Supervision: BM. All authors contributed to the article and approved the submitted version.
